# Alchemix, p53 and topoisomerase

**DOI:** 10.18632/aging.100803

**Published:** 2015-09-17

**Authors:** Roger J Grand, Grant S Stewart

**Affiliations:** School of Cancer Sciences, College of Medicine and Dentistry, University of Birmingham, Birmingham, U.K. B15 2TT

**Keywords:** Alchemix, chemotherapy, DNA damage response, chemotherapy, topoisomerase

As our knowledge of the DNA damage response (DDR) has increased, our understanding of the underlying causes of many cancers has become clearer. It is now apparent that DDR genes constitute a significant subset of tumour suppressor genes, with defects in their signalling pathways associated with a wide variety of cancers [1]. Importantly, as well as allowing us to recognize the aetiology of cancers, greater insight into the DDR has helped in the design of novel cancer therapies. For example, the use of PARP1 inhibitors for the treatment of malignancies lacking functional *BRCA1* or *BRCA2* genes has opened up a novel means of combatting certain breast and ovarian tumours [2, 3]. Initial attempts to design specific anti-cancer drugs, based on intervention in DDR pathways, have relied on the induction of DNA damage and subsequent activation of apoptosis through the p53 pathway [4]. For example, fludarabine and cisplatin fall into this category. As it has become clear that activation of the DDR is a very effective approach to killing tumours, a substantial amount of time, money and effort has been devoted to developing more specific ways of activating the DDR [4].

One class of compounds that is frequently used as anti-cancer agents are topoisomerase II (Top 2) inhibitors, which are chemically based on a planar tricyclic pharmacophore [5]. Examples of such drugs are doxorubicin, daunorubicin and mitoxantrone. These compounds act as Top 2 poisons and ‘trap’ topoisomerase II-DNA cleavage complexes such that DNA double strand breaks generated cannot be re-ligated. Top 2 poisons, however, suffer from the drawback that they are relatively ineffective in tumours that express the multi-drug resistance genes or lack sufficient Top 2 expression. Furthermore, mutations in other DDR genes such as *ATM* and *TP53* result in tumours becoming refractory to Top 2 inhibitors. In an attempt to combat this, a class of drugs has been developed, based on disubstituted non-symmetric anthraquinones, which combines DNA intercalating and alkylating activities [6]. Alchemix has previously been reported to be the most potent of these compounds, with an ability to have anti-cancer activity against both doxorubicin and cisplatin resistant tumour xenografts in mice [6].

In a recent publication the mode of action of Alchemix has been described in detail [7]. The drug is capable of inducing a DDR at nanomolar concentrations and this is due to activation of ATR- and DNA-PK-dependent pathways. Both the alkylating and Top 2 inhibitory activities of Alchemix contribute to this. Despite the generation of double strand breaks after prolonged exposure to Alchemix, there is little evidence of ATM activation as determined by a lack of ATM auto-phosphorylation and ATM-dependent phosphorylation of Chk2 or the rapid formation of γH2AX/53BP1 foci. The observation that compromising the level or activity of ATR markedly reduces the cellular DDR response to Alchemix indicates that, unlike other intercalating Top 2 inhibitors, it elicits an ATR- rather than an ATM-dependent DDR.

In view of topoisomerase II's involvement in DNA unwinding prior to anaphase it is to be expected that Alchemix would have a detrimental effect on cell cycle progression through S phase. In response to low doses of the drug it was shown that a proportion of epithelial cells accumulate in S phase. However, the remainder of the cells bypass the checkpoint and go into mitosis with damaged DNA, resulting in cells with misaligned and fragmented chromosomes and multipolar spindles. These cells die, not by apoptosis or autophagy, but probably by mitotic catastrophe or abortive mitosis. Unsurprisingly, cell death occurring via this mechanism is unaffected by the p53 status. At very high doses of Alchemix (>1μM) limited apoptosis is observed to occur in cells expressing *wt* p53, although this appears to represent a minor route by which cells die following Alchemix treatment. Through further study it is apparent that rapidly cycling cells and those undergoing replication stress due to oncogene expression, are particularly susceptible to killing by Alchemix and, again, this is not due to induction of apoptosis but occurs, most probably, via mitotic catastrophe.

To confirm the ability of Alchemix to function *in vivo*, its action against engrafted T-cell acute lymphoblastic leukaemia (T-ALL) cell lines in NOG mice was investigated. The drug caused marked reduction in the size of subcutaneous tumours. Similarly, when a primary paediatric T-ALL sample was engrafted into the mice, Alchemix injection led to a substantial reduction in spleen size and the presence of human CD45 expressing tumour cells in the mouse spleens.

Resistance of a proportion of tumour cells to treatment is one of the major drawbacks of chemotherapy. This is often attributable to mutation in one or more DDR genes. Significantly, some of the T-ALL cell lines used in this study express mutant p53. However, this did not affect the ability of Alchemix to kill the tumour cells *in vitro* or cause a reduction in tumour load *in vivo*. Surprisingly, it was noted that death of T-ALL cell lines induced by Alchemix is primarily due to the induction of apoptosis rather than mitotic catastrophe, although again this occurs independently of p53. In other studies Alchemix has been shown to be effective against engrafted human ovarian cell lines which had developed resistance to cisplatin or doxorubicin [6]. The resistance to doxorubicin was, at least partially, due to the overexpression of P-gp drug efflux pump and the absence of *MLH1*. This mechanism of resistance has been associated with other tumour types in addition to ovarian cancer. The ability of Alchemix to cause regression of these tumours *in vivo* is consistent with its ability to elicit a potent DDR in MSH-depleted cells [7] or cells over-expressing the MDR1 gene.

In summary, Alchemix represents a novel chemotherapeutic reagent which is able, at low doses, to induce death in tumour cells by mitotic catastrophe and/or apoptosis. Through its ability to activate different DDR pathways it has the potential to be used as a therapeutic agent against tumours which have mutations in DDR genes, such as *p53* and *ATM*, and are therefore refractory to more commonly used agents.
